# Tissue Inhibitor of Metalloproteinases-3 Peptides Inhibit Angiogenesis and Choroidal Neovascularization in Mice

**DOI:** 10.1371/journal.pone.0055667

**Published:** 2013-03-01

**Authors:** Jian Hua Qi, Quteba Ebrahem, Mariya Ali, Alecia Cutler, Brent Bell, Nicholas Prayson, Jonathan Sears, Vera Knauper, Gillian Murphy, Bela Anand-Apte

**Affiliations:** 1 Department of Ophthalmology, Cole Eye Institute, Cleveland Clinic Lerner College of Medicine at Case Western Reserve University, Cleveland, Ohio, United States of America; 2 Dept. of Molecular Medicine, Cleveland Clinic Lerner College of Medicine at Case Western Reserve University, Cleveland, Ohio, United States of America; 3 Metalloproteinase Research Group, Matrix Biology and Tissue Repair Research Unit, Dental School, Cardiff University, Heath Park, Cardiff, Wales, United Kingdom; 4 Cancer Research United Kingdom Cambridge Research Institute, The Li Ka Shing Centre, Robinson Way, Cambridge, United Kingdom; Institut de la Vision, France

## Abstract

Tissue inhibitors of metalloproteinases (TIMPs) while originally characterized as inhibitors of matrix metalloproteinases (MMPs) have recently been shown to have a wide range of functions that are independent of their MMP inhibitory properties. Tissue inhibitor of metalloproteinases-3 (TIMP-3) is a potent inhibitor of VEGF-mediated angiogenesis and neovascularization through its ability to block the binding of VEGF to its receptor VEGFR-2. To identify and characterize the anti-angiogenic domain of TIMP-3, structure function analyses and synthetic peptide studies were performed using VEGF-mediated receptor binding, signaling, migration and proliferation. In addition, the ability of TIMP-3 peptides to inhibit CNV in a mouse model was evaluated. We demonstrate that the anti-angiogenic property resides in the COOH-terminal domain of TIMP-3 protein which can block the binding of VEGF specifically to its receptor VEGFR-2, but not to VEGFR-1 similar to the full-length wild-type protein. Synthetic peptides corresponding to putative loop 6 and tail region of TIMP-3 have anti-angiogenic properties as determined by inhibition of VEGF binding to VEGFR-2, VEGF-induced phosphorylation of VEGFR-2 and downstream signaling pathways as well as endothelial cell proliferation and migration in response to VEGF. In addition, we show that intravitreal administration of TIMP-3 peptide could inhibit the size of laser-induced choroidal neovascularization lesions in mice. Thus, we have identified TIMP-3 peptides to be efficient inhibitors of angiogenesis and have a potential to be used therapeutically in diseases with increased neovascularization.

## Introduction

Tissue inhibitors of metalloproteinases (TIMPs) constitute a family of four proteins (TIMP-1, TIMP-2, TIMP-3 and TIMP-4) that are endogenous inhibitors of matrix (MMP) and play a critical role in the maintenance of extracellular matrix (ECM) homeostasis. In general, all four TIMPs are broad-spectrum inhibitors of the MMP family, with some differences in specificity. TIMP-3 has been demonstrated to have a broader range of metalloproteinase substrates being particularly effective in uniquely inhibiting several members of the ADAM (a disintegrin and metalloprotease) and ADAMTS (ADAM with thrombospondin motifs) family [1], [2], [3], [4], [5], [6]. Although originally characterized for their functional property to inhibit MMP activity, TIMPs have more recently been shown to have additional biological activities that may be independent of their MMP-inhibitory functions [7]. We have previously demonstrated that TIMP-3 is a potent angiogenesis inhibitor, and functions independently of its MMP inhibitory activity in this regard, by blocking the binding of vascular endothelial growth factor (VEGF) to its receptor VEGFR-2 [8].

The three–dimensional structure analysis of TIMP-1 and TIMP-2 revealed by X-ray crystallography identified the presence of two distinct domains; a 125 amino acid N-terminal domain and a 65 amino acid C-terminal domain, each stabilized by three disulfide bonds [9]. In addition to an oligonucleotide and oligosaccharide binding fold, the N-domain (which contains the MMP inhibitory activity) contains a five-stranded closed twisted β-barrel with a greek key topology and three α-helices. The C-domain contains a pair of parallel β strands associated with a loop followed by a helix and a pair of antiparallel β strands linked by a β-hairpin [10]. To identify the anti-angiogenic functional domains of TIMP-3, we performed a series of structure-function analyses examining VEGF binding to VEGFR-2, and downstream endothelial cell proliferation and migration. We determined that the COOH-terminal domain of TIMP-3 contains the angio-inhibitory activity with the NH2-terminal domain being inert for this function. We further mapped the ability to block VEGF binding to VEGFR-2 to the “loop 6” and “tail” peptides and the capability of “loop 6” to inhibit choroidal neovascularization (CNV) in a rodent model.

## Materials and Methods

### Materials

Porcine Aortic Endothelial cells expressing VEGFR-2 (PAE_KDR_) were cultured in Ham’s F-12/DMEM medium supplemented with 10% fetal bovine serum (FBS) (Hyclone), 50 units/ml penicillin and 50 µg/ml streptomycin as described previously [11]. Recombinant human VEGF was a kind gift from Genentech, CA. Antibodies: Anti-Phosphotyrosine clone 4G10 (Upstate Biotechnology/Millipore, Billerica, MA), monoclonal anti-Flk-1 (A-3) (Santa Cruz Biotechnology, Santacruz, CA), MAPK and phospho-specific MAPK antibodies (Calbiochem-Novabiochem Corporation/EMD chemicals, Gibbstown, NJ).

### Generation and Purification of Recombinant TIMP-3 Proteins

Full length, recombinant human TIMP-3 was purified from stably transfected mouse myeloma cells as described previously [12]. The human N-TIMP-3 expression vector was designed as fusion protein encompassing amino acids 1–115, with an extension of 25 amino acids at the C-terminus (Val-Asp-Ala-Ala-Ala-Glu-Gln-Lys-Leu-Ile-Ser-Glu-Glu-Asp-Leu-Asn-Gly-Ala-Ala–His-His-His-His-His-His) and the protein purified and refolded following transformation of ultracompetent E.coli BL21(DE3) as described previously [13].The N-TIMP2/C-TIMP-3 chimera was constructed using overlapping extension PCR. The amplified N terminal domain of TIMP-2 (residues 1–127) and the C-terminal domain of TIMP-3 (residues 122–188) were combined and subjected to PCR using a forward primer complementary to the N-TIMP-2 and a reverse primer complementary to the end of C-TIMP-3. The resulting N-TIMP-2-C-TIMP-3 cDNA was cloned into BL21(DE3)pLysS E. coli and large scale cultures grown as described for N-TIMP-3 [13]. Following ITPG (Isopropyl-β-D-thio-galactoside) induction, inclusion bodies were sheared in Tris buffered saline, 1%Tween-20 and sonicated. Subsequent to centrifugation, the pellets were washed in 1 M urea and ddH2O. After protein concentration estimations, the protein was suspended in solubilization buffer and then subjected to a refolding protocol with refolding buffer (0.45 M GuHCl, 100 mM Tris-HCl, pH 8.75, 0.8 mM GSH, 0.45 mM GSSG) and allowed to stir overnight at 4°C. The refolded protein was dialysed against 2 changes of 40 L of 10 mM acetate, pH 6.0 and centrifuged (15 min, 10000 rpm) to remove precipitate. The cleared protein solution was loaded under gravity onto a 30 ml SP-Sepharose column (Sigma S-1799) pre-equilibrated in 10 mM Na Acetate, pH 6.0. The column was washed with 3 volumes of the same buffer and the protein eluted using 50 mM Tris-HCl, pH 7.5, 0.5 M NaCl, 20%glycerol. Samples of purified protein were compared under reducing and non-reducing conditions by combining the protein with the appropriate sample buffer and loading them on a 12.5% SDS-PAGE gel with MW markers. N-TIMP-2-C-TIMP-3 was active-site titrated against MMP2, with total protein concentrations estimated using A280 readings.

### Peptide Synthesis

The peptides were synthesized by Fmoc strategy on an Omega 396 synthesizer (Advanced ChemTech, Louisville, KY) using solid phase chemistry. Preloaded Fmoc-Wang resin and Fmoc-L-amino acids were procured from Anaspec (San Jose, CA). The peptide synthesis was performed by double coupling amino acid esters of 1-hydroxybenzotriazole (HOBt) using 1,3 diisopropylcarbodiimide (DIC) as the coupling agent. A six-fold excess of N-alpha-Fmoc amino acid esters of HOBt in NMP were used in the synthesis. A 1∶1 ratio of amino acid to DIC was used in all the coupling reactions. Deprotection of N-alpha-Fmoc group was accomplished by 25% piperidine in dimethylformamide twice; first for 5 minutes and than a second time for 25 minutes. After the synthesis was completed, the peptides were cleaved from the solid support and deprotected using a modified reagent K(r) cocktail consisting of 88%TFA, 3%thioanisole, 5% ethanedithiole, 2%water and 2% phenol. Four mls of cleavage cocktail was added to the dried peptide-resins in a 15 ml glass vial blanketed with nitrogen and cleavage was carried out for 2.5 hrs with gentle magnetic stirring. At the end cleavage time, the cocktail mixture was filtered on a Quick-Snap column. The filtrate was collected in 20 ml ice-cold butane ether. The peptides were allowed to precipitate for an hour at −20°C, centrifuged, and washed twice with ice-cold methyl-t-butyl ether. The precipitate was dissolved in 25% acetonitrile and lyophilized to complete dry powder.

Quality of peptides was analyzed by analytical reverse phase HPLC and MALDI-TOF-TOF (matrix assisted laser desorption ionization time-of-flight) Mass Spectrometer, model 4800 from Applied Biosystems. The peptide was purified on TARGA C18 semi-preparative column (250×10 mm) from Higgins Analytical Inc.,USA.

Peptide sequences were designed to represent various smaller structural domains of the carboxyl terminus of TIMP-3. These include: a 10-amino acid peptide corresponding to loop 5 with sequence (KIKSCYYLPCFVTS), a 24-amino acid peptide corresponding to loop 6 with sequence (KNECLWTDMLSNFGYPGYQSKHYACIRQKG), and a 19-amino acid peptide corresponding to the carboxy-terminal tail with sequence (GYCSWYRGWAPPDKSIINATDP). A fourth peptide of sequence (RGFTKMPHVQYIHTEASESL) corresponding to a conserved sequence in the NH2-terminal domain of all TIMPs was also synthesized.

### Competitive ELISA

96 well EIA/RIA strip plates (COSTAR) were coated with 80 ng/ml recombinant VEGF overnight at 4°C, washed and blocked with blocking buffer (1% BSA and 5% sucrose) overnight at 4°C. Series of dilutions were prepared for recombinant proteins representing WT-TIMP-3, NT2:CT3 and N-T3 in PBS or synthetic peptides corresponding to T3-loop 5, T3-loop 6, T3-N or T3-tail peptides in 0.5 M Tris-HCl (pH 7.5). The diluents were mixed with 50 ng/ml recombinant human VEGFR-2 (KDR/Fc chimera, recombinant human VEGFR-1 (Flt-1)/Fc chimera (R&D Systems, Minneapolis, MN) or control human lgG (Sigma Aldrich, St Louis, MO). The mixtures were then added to the VEGF-coated well in a final volume of 100 µL in PBS and incubated for 2 hours at room temperature. Bound VEGFR-2 was detected by HRP-conjugated anti-human lgG(Fc specific) (1∶1000).

### Immunoprecipitation and Immunoblotting

Immunoprecipitates of cell lysates with the indicated antibodies were subjected to SDS-PAGE and western blot analysis. Proteins were detected with either a HRP-conjugated anti-rabbit or anti-mouse IgG antibody (Amersham Pharmacia Biotech., Piscataway, NJ) followed by ECL. The blots were restripped with Western ReProbe™ solution (GBiosciences, Maryland Heights, MO) for 30 minutes and reprobed as indicated.

### Immunofluorescence Staining Analyses

Subconfluent cells cultured on Falcon culture slides (BD Biosciences) were serum-starved overnight and stimulated for 30 min at 37°C with 50 ng/ml VEGF or control buffer. The cells were fixed with 3.7% paraformaldehyde in PBS for 30 min at 4°C and permeabilized with 0.2% Triton X-100 for 20 min at room temperature. Following rinsing with PBS, the cells were incubated with fluorescein isothiocyanate (FITC)-labeled phalloidin in PBS (0.66 µg/ml, Sigma Aldrich, St. Louis, MO) for 20 min at room temperature, washed with PBS and analyzed by fluorescence microscopy.

### Migration Assay

A modified Boyden chamber assay was performed as described previously [8]. Briefly, 8.0 µm pore PVPF polycarbonate membranes were pre-coated with 100 µg/ml collagen type I in 0.2 N acetic acid (Cohesion technologies, Inc. Palo Alto, California). VEGF at the indicated concentrations was placed in the lower chamber, and cells (2×10^5^) in serum-free medium were placed in the upper chambers. The chamber was then incubated for 4 hrs at 37°C in a 5% CO_2_, humidified incubator. Cells remaining on the top of the filter were removed. Cells on the bottom surface of the filter were fixed (in 10% formalin), stained (Hematoxilin) and mounted (in Permount). The number of migrating cells per well was evaluated by light microscopy.

### Proliferation Assay

Cells were seeded at a density of 2000 cells/well in 24 well culture plates and cultured for 24 h in growth medium. After synchronization overnight in serum-free medium supplemented with 0.1% BSA, the cells were stimulated with or without VEGF for 36 h in the presence or absence of the indicated concentrations of TIMP-3 peptides. After a 5-day incubation, cells were trypsinized and counted using a Coulter particle counter.

### Laser-induced CNV Assay

All animal studies were approved by the Animal Care and Use Committee of the Cleveland Clinic (IACUC number ARC-08792) and conformed to the National Institutes of Health Guide for the Care and Use of Animals in Research and the ARVO statement for the use of animals in ophthalmic and vision research. All surgery was performed under ketamine/xylazine anesthesia with maximum effort devoted to minimizing suffering. Laser photocoagulation-induced rupture of Bruch’s membrane was used to generate CNV as previously described [14]. Briefly, 4–5 week old C57BL/6J mice were anesthetized with ketamine hydrochloride (100 mg/kg body weight) and xylazine (10 mg/kg body weight) followed by1% Tropicamide (Alcon laboratories, Inc., Fort Worth, TX) for pupillary dilation. Three burns of 532-nm diode laser photocoagulation (Oculight; Iridex, Mountain View California; 50-µm spot size, 0.1 second duration, 200 mW) were delivered to each retina using a slit lamp delivery system and a hand held coverslip as a contact lens. Burns were performed in the 9, 12 and 3 o’clock positions of the posterior pole of the retina. Production of a bubble at the time of lasering indicated a successful burn. At the same time, intravitreal injections of TIMP-3 peptides or control PBS was performed. Two weeks later, mice were anesthetized and perfused with fluorescein-labeled dextran (2×10^6^ average molecular weight, Sigma Aldrich), choroidal flatmounts were prepared and CNV area measured. For the experiments using tail peptide, the animals were visualized by confocal scanning laser ophthalmoscope (cSLO, HRA2, Heidelberg Engineering, Inc.) following Fluorescein angiography(FA) The imaging field-of view was 55 degrees.

Five mice were used for each dose of peptide, with three burns in each eye (n = 15–18 successful burns in each group). For quantitative analysis of lesion intensity and size, CNV images were batch processed using a custom macro generated in Image-Pro Plus 5.1 (Media Cybernetics, Silver Spring, MD). For each image, a region of interest (ROI) was traced around the lesion using a wand tool (a manual trace was performed in a few cases where the lesion was not significantly brighter than the background). Mean intensity (range between 10–255 gray levels), perimeter, area, and mean diameter (pixels) were calculated for each ROI and exported to excel. Analyses were performed in a blinded fashion to eliminate user bias.

### Statistical Analysis

Data are presented as mean ± SD. The statistical significance of differential findings observed between experimental and control groups were determined using one-way analysis of variance (ANOVA) and considered to be significant if P values were <0.05.

## Results

### COOH-terminal TIMP-3 Domain Inhibits VEGF Binding to VEGFR-2

**Figure 1:**
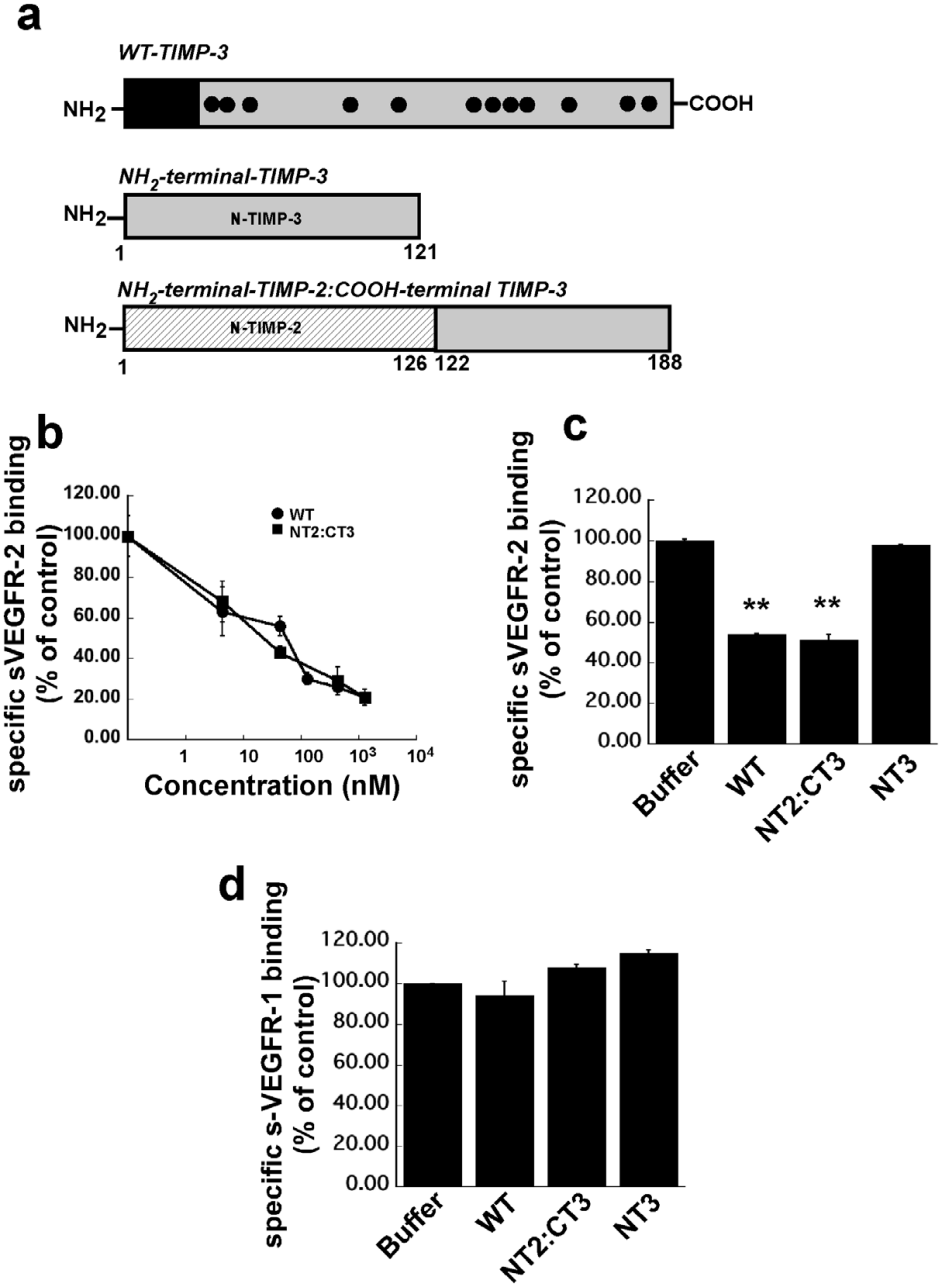
The effects of recombinant wild type TIMP-3 (WT-TIMP-3), N-terminal TIMP2: C-terminal TIMP-3 chimera (NT2: CT3) and the NH3-terminal domain of TIMP-3 (NT3) on binding of 50 ng/ml soluble(s) VEGFR-1 or VEGFR-2 to immobilized VEGF. A competitive ELISA assay was used as described under “Materials and Methods”. (a) description of WT-TIMP-3, N-T3 and NT2:CT3 (b). WT-TIMP-3 and NT2: CT3 inhibit sVEGFR-2-VEGF complexes in a concentration-dependent manner. (c). N-T3 has no effect on sVEGFR-2-VEGF complexes. (d). WT-TIMP-3, NT2 and N-T3 are unable to block sVEGFR-1-VEGF complexes.

NH2 terminal TIMP-3 protein [N-T3] (residues 1–121) and an NH2-terminal TIMP-2: COOH-terminal TIMP-3 chimera [NT2:CT3] representing the isolated C-terminal domain of TIMP-3 (residues 1–126 of TIMP-2 and residues 122–188 of TIMP-3) (Fig. 1a) were purified as described previously [15].

We performed a competitive ELISA to test the ability of WT TIMP-3, N-T3 and NT2:CT3 to block the binding of VEGF to its receptors VEGFR-2 and VEGFR-1. Soluble VEGFR-2/Fc chimera (sVEGFR-2/Fc chimera) bound to VEGF immobilized on ELISA plates in a concentration dependent manner (data not shown). In the presence of recombinant WT-TIMP-3 protein and NT2:CT3, the binding of VEGF to sVEGFR-2/Fc chimera was markedly decreased (IC50 value of 41 nM and 28 nM respectively) (Fig. 1b). In contrast, the addition of recombinant N-T3 did not disrupt the formation of VEGF-VEGFR-2 complexes (Fig. 1c) indicating that the anti-angiogenic activity of TIMP-3 is present in the COOH-terminal domain. In addition, the ability to block the binding of VEGF to its receptor is specific for VEGFR-2 as neither full length WT-TIMP-3 nor COOH-terminal TIMP-3 blocked the binding of VEGF to VEGFR1 (sFlt-1) (Fig. 1d).

### Preparation of COOH-terminal TIMP-3 Peptides

To further map the anti-angiogenic activity of TIMP-3 within its COOH-terminal domain, four peptides corresponding to various smaller domains of T3 were synthesized. COOH-terminal peptides of TIMP-3 were prepared by solid-phase peptide synthesis using Fmoc-protected amino groups. The final purification was performed using a TARGA-C18 semi-preparative column and electrospray ion trap mass spectrometry was used to determine the average molecular mass (M_r_). NH2-terminal sequencing in addition to MS confirmed the purity and molecular mass of the peptides. The M_r_s and sequences of the purified peptides are indicated in Table 1.

### T3-loop 6 and T3-tail Peptides Inhibit the Binding of VEGF to sVEGFR-2

**Figure 2:**
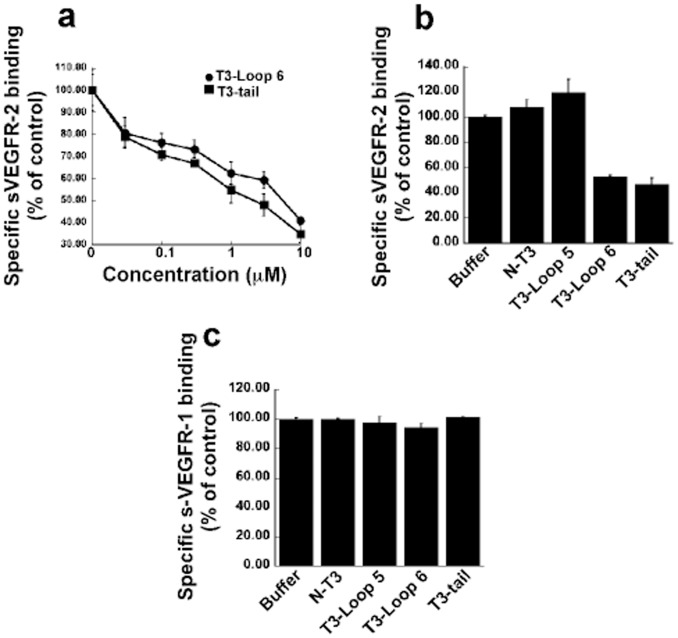
The effect of synthetic TIMP-3 peptides on binding of 50 ng/ml soluble (s) VEGFR-1 or –2 to immobilized VEGF. A competitive ELISA assay was used as described under “Materials and Methods” (a). Loop 6 and tail peptides inhibit sVEGFR-2-VEGF complexes in a concentration-dependent manner. (b) Loop5 and N-peptide have no effect on sVEGFR-2-VEGF complexes. (c) Loop 5, loop 6, tail and N-peptide are unable to inhibit sVEGFR-1-VEGF complexes.

VEGF-A signals primarily via the VEGF-receptor tyrosine kinases, VEGFR-1 and 2, with VEGFR-2 being implicated as the predominant pro-angiogenic receptor. The role of VEGFR-1 has been more controversial with some data demonstrating it as having a pro-angiogenic role [16] and others an anti-angiogenic function [17]. The T3-peptides were initially tested for their ability to block the binding of VEGF to sVEGFR-2/Fc chimera in an *in vitro* competitive ELISA. T3-loop 6 and T3-tail could inhibit binding of VEGF to sVEGFR-2/Fc chimera (Fig. 2a) in a concentration dependent fashion with an IC50 of 2 and 5 µM respectively. In contrast, two peptides corresponding to T3-loop 5 and the NH2-terminal domain of intact TIMP-3 (N-peptide) did not show any inhibitory activity (Fig. 2b). Interestingly, the IC 50 of the small peptides was in the µM range compared with the nM range doses seen with the entire COOH-terminal T3 domain. In addition, as observed with the COOH-terminal T3 domain, T3-loop 6 and T3-tail did not inhibit VEGF binding to VEGFR-1 (Flt-1) (Fig. 2c). Whether pan-inhibition of VEGF is necessary for inhibition of neovascularization or if inhibition of signaling via VEGFR-2 is sufficient has not been ascertained. Based on our results with TIMP-3 peptides we postulated that the TIMP-3 would be a useful tool to determine if specific inhibition of binding of VEGF to VEGFR-2 could inhibit VEGF-mediated angiogenesis.

### T3-loop 6 and T3-tail Peptides Inhibit VEGFR-2 Signaling in Endothelial Cells

**Figure 3:**
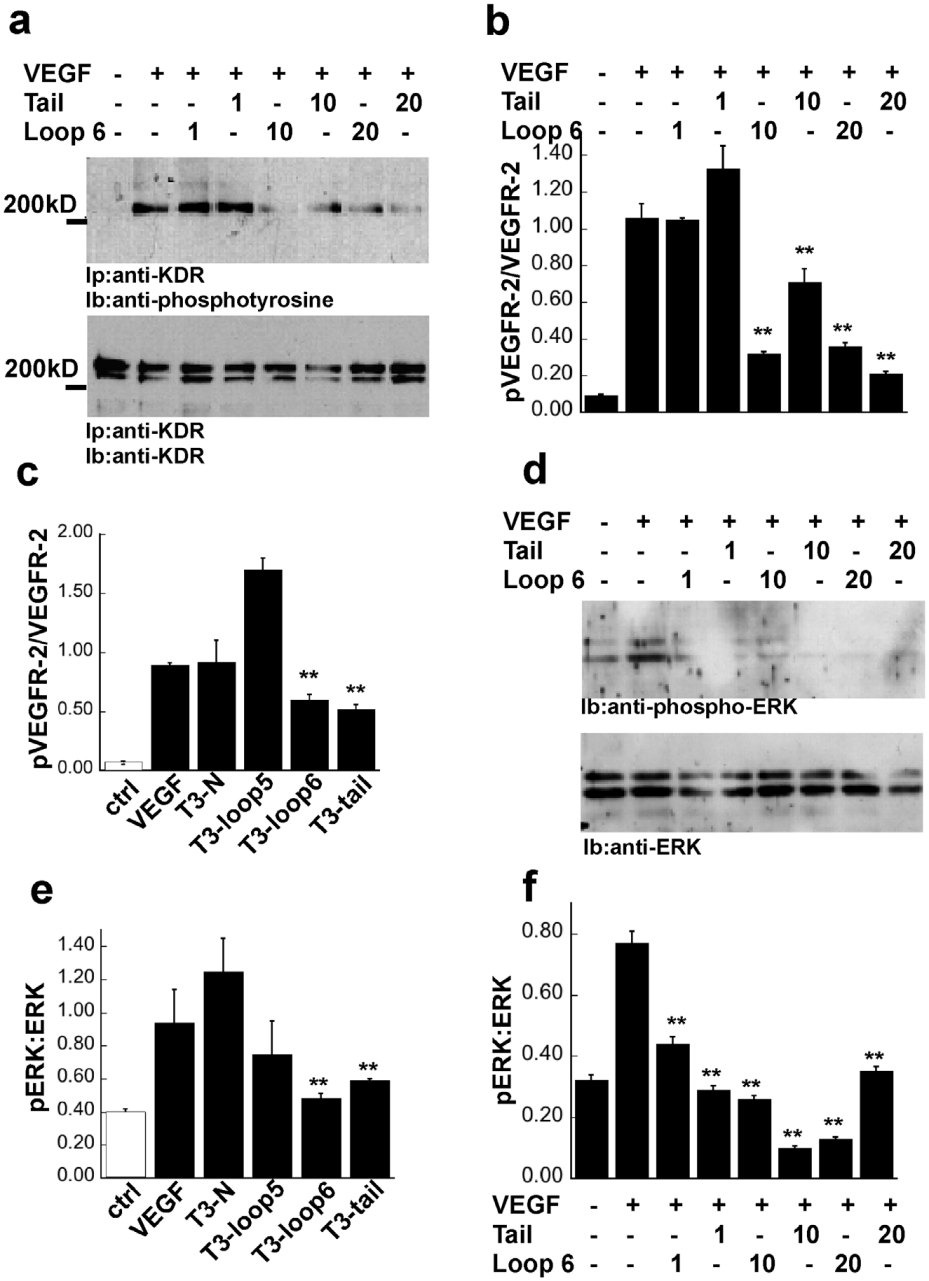
Effects of TIMP-3 peptides on VEGF signaling in endothelial cells. (a) TIMP-3 loop 6 and tail inhibit VEGF-mediated phosphorylation of VEGFR-2 in PAE/KDR cells in a concentration dependent manner. Prior to treatment with indicated concentrations of TIMP-3 peptides for 30 min, the serum-starved cells were stimulated or not with 50 ng/ml VEGF for 10 min in the presence or absence of the same concentration of peptide. Cell lysates were separated by SDS-PAGE (10%) and analyzed by immunoprecipitation with an anti-KDR anti-body followed by immunoblotting with an anti-phosphotyrosine antibody, 4G10 (top panel). KDR protein was analyzed by immunoprecipitation and immunoblotting of cell lysates using anti-KDR antibody (bottom panel). (b) Densitometric quantitation of 210-kDa bands for phospho- or total mature form of KDR. (c) TIMP-3 loop 6 and tail but not loop 5 and N-peptide inhibit VEGF-mediated phosphorylation of KDR in PAE/KDR cells. (d) TIMP-3 loop 6 and tail inhibit ERK phosphorylation. Phosphorylation of ERK1 and ERK2 in response to VEGF was detected by immunoblotting with phosphospecific MAP kinase antibodies (top panel). Total protein levels of ERK was determined by immunoblotting with anti- MAP kinase antibodies (bottom panel). (e) TIMP-3 loop 6 and tail but not loop 5 nor N-peptide inhibit VEGF-mediated phosphorylation of ERK in PAE/KDR cells.(f) Densitometric quantitation of pERK (top panel of d) and ERK bands (bottom panel of d) for phospho- or total ERK proteins. **significantly different from control (without peptides+VEGF), p<0.01 (Student’s t test).

We examined whether T3-loop 6 and T3-tail could regulate signaling events that follow VEGF binding to VEGFR-2 in endothelial cells (PAE/KDR). Prior to treatment with the T3-peptides at various concentrations, serum-starved endothelial cells were stimulated with VEGF. Immunoprecipitation of VEGFR-2 followed by western blot analysis with anti-phosphotyrosine antibodies was performed to evaluate the autophosphorylation of VEGFR-2 by VEGF in the presence of T3 peptides. As shown in Fig. 3a, a mature form of VEGFR-2 corresponding to a molecular size of 210-kDa was phosphorylated in control cells following treatment with VEGF. Treatment of endothelial cells with 10 µM T3-loop 6 or T3-tail peptide resulted in a significant inhibition of VEGF-induced receptor autophosphorylation (Fig. 3a, upper panel). Western blot analysis of the same blot with antibodies directed against the C-terminus of VEGFR-2, determined that there was no effect of the peptides on VEGFR-2 levels on the surface of endothelial cells (Fig. 3a, lower panel). To quantitate phosphorylation of VEGFR-2, densitometry of the phosphorylated VEGFR-2 band (pVEGFR-2) as well as the total VEGFR-2 band (KDR) was performed. The ratio of pVEGFR-2:VEGFR-2 was determined and compared between peptide treatments (Fig. 3b). 20 µM T3-loop 5 or T3-N peptide failed to inhibit VEGF-induced receptor activation (Fig. 3c).

Activation of MAP kinase is a known VEGFR-2 downstream signaling event. We analyzed the effect of TIMP-3 peptides on the phosphorylation of MAP kinases, ERK1 (p44) and ERK2 (p42), in endothelial cells exposed to VEGF by western blot analysis. Fig. 3d shows T3-loop 6 or T3-tail peptides inhibited VEGF-stimulated phosphorylation of ERK1/2 (Fig. 3d,f). In contrast, neither loop 5 nor N-peptide, even at higher concentrations (20 µM) inhibited VEGF-stimulated MAP kinase activation (Fig. 3e).

### T3-Loop 6 and T3-tail Peptides Inhibit VEGF-stimulated Endothelial Cell Migration and Actin Reorganization of PAE/KDR Cells

**Figure 4:**
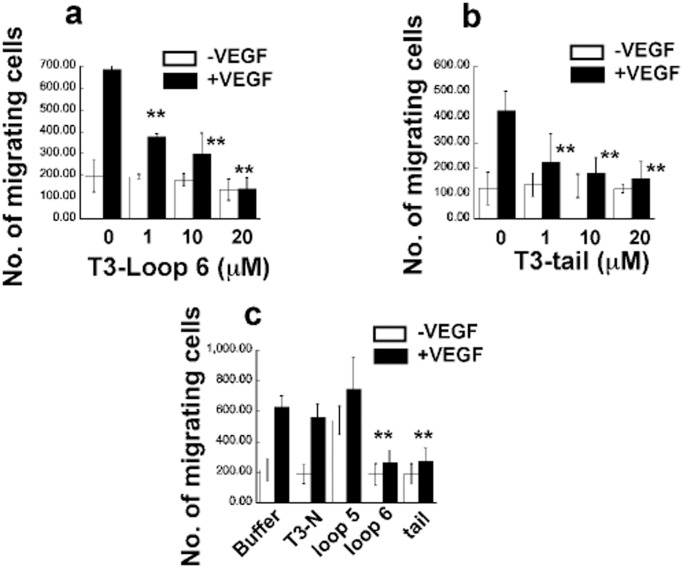
Effects of TIMP-3 peptides on VEGF-mediated EC migration. TIMP-3 loop 6 and tail but not loop 5 and N-peptide inhibit VEGF-induced migration in PAE/KDR cells. Cells were analyzed for migration towards 50 ng/ml VEGF in the presence or absence of increasing concentrations of loop 6 (a) and tail peptides (b) or 20 µM loop 5 or N-peptide (c), respectively, using a mini-Boyden chamber incubated at 37°C for 4 h. Migrating cell number is expressed as means± SEM of quadruplicate samples. **significantly different from control (without peptides), p<0.01 (Student’s t test).

Migration of endothelial cells is essential for angiogenesis. We tested the TIMP-3 peptides for their ability to block chemotaxis of endothelial cells to VEGF using a Boyden mini-chamber assay. T3-loop 6 (Fig. 4a) and T3-tail (Fig. 4b) significantly inhibited VEGF-induced migration at doses of 1, 10 and 20 µM. Interestingly, basal migration in the absence of VEGF was not inhibited by these peptides. In contrast, loop 5 and N-peptide at doses of up to 20 µM failed to inhibit VEGF-induced migration (Fig. 4c).

**Figure 5:**
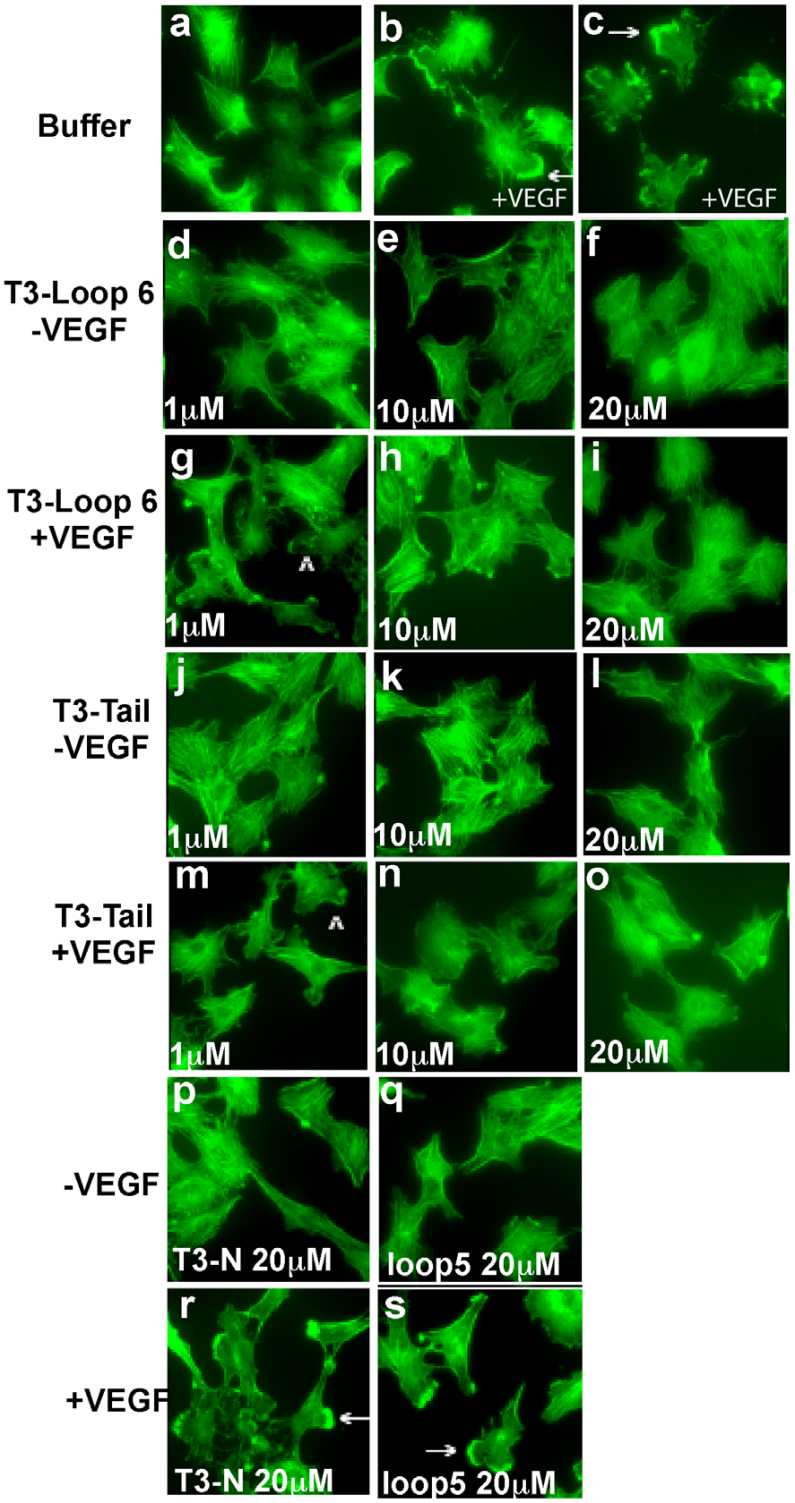
Effects of TIMP-3 peptides on VEGF-mediated actin reorganization. TIMP-3 loop 6 and tail but not loop 5 and N-peptide inhibit VEGF-induced actin reorganization in PAE/KDR cells. Quiescent cells were stimulated with (b,c,g,h,i,m,n,o,r,s) or without (a,d,e,f,j,k,l,p,q) 50 ng/ml VEGF in the presence or absence of increasing concentrations of loop 6 (d-i) and tail peptides (j-o) or 20 µM loop 5 (q,s) or N-peptides (p,r) for 30 min at 37°C. Cells were permeabilized and stained with fluorescein isothiocyanate-phalloidin, as described under “Materials and Methods”. The arrows indicate membrane edge ruffling in the stimulated cells. Magnification, ×1000.

Endothelial cell migration involves reorganization of the actin cytoskeleton with the formation of membrane edge ruffles, that have previously been shown to be an integral part of cell motility responses. We analyzed the effects of the TIMP-3 peptides on VEGF-stimulated formation of membrane ruffles, in endothelial cells. As previously described [18], membrane ruffles were induced in a majority of endothelial cells following VEGF stimulation for 30 min (Fig. 5b and 5c). Pretreatment with T3-loop 6 or T3-tail peptides at doses of 10 µM and 20 µM significantly inhibited the VEGF-stimulated induction of membrane ruffles (Figs. 5h, 5i, 5n, 5o). In contrast, neither T3-loop 5 nor T3-N peptide at doses as high as 20 µM inhibited the formation of membrane ruffles (Fig. 5r and 5s).

### T3-loop 6 and T3-tail, but not T3-loop 5 Peptides Inhibit the VEGF-induced Proliferation of Endothelial Cells

**Figure 6:**
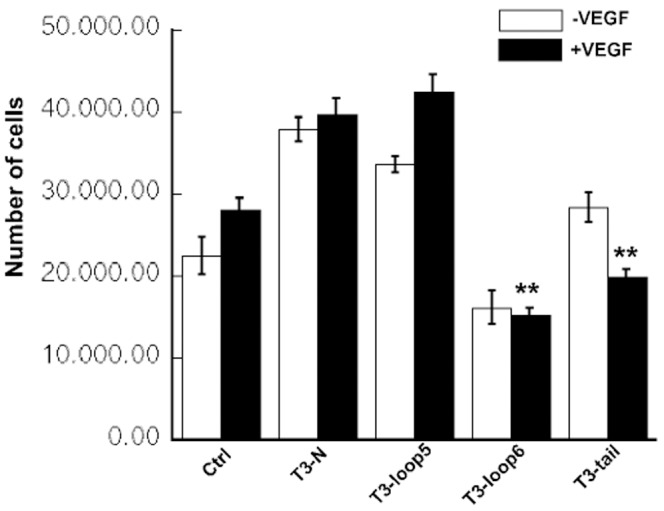
Effects of TIMP-3 peptides on VEGF-mediated EC proliferation. TIMP-3 loop 6 and tail but not loop 5 and N-peptide inhibit VEGF-induced proliferation in PAE/KDR cells. Serum-starved cells were incubated with or without VEGF (5 ng/ml) in the presence or absence of the 1 µM of TIMP-3 peptides for 5 days. Cell numbers were counted using a Coulter particle counter. **significantly different from control (with VEGF), p<0.01 (Student’s t test).

Proliferation of endothelial cells is a critical component of angiogenesis. We examined the ability of TIMP-3 peptides to suppress VEGF-induced proliferation of endothelial cells. Quiescent cells were incubated with VEGF in the presence or absence of peptides. We determined that T3-loop 6 and T3-tail peptides inhibited VEGF-stimulated endothelial cell proliferation at doses as low as 1 µM. Under similar conditions, neither T3-loop 5 nor T3-N peptide showed inhibitory properties(Fig. 6) and may in fact induce proliferation of endothelial cells.

### T3-loop 6 and T3-tail Peptides Inhibit Choroidal Neovascularization (CNV) in an *in vivo* Mouse Model

**Figure 7:**
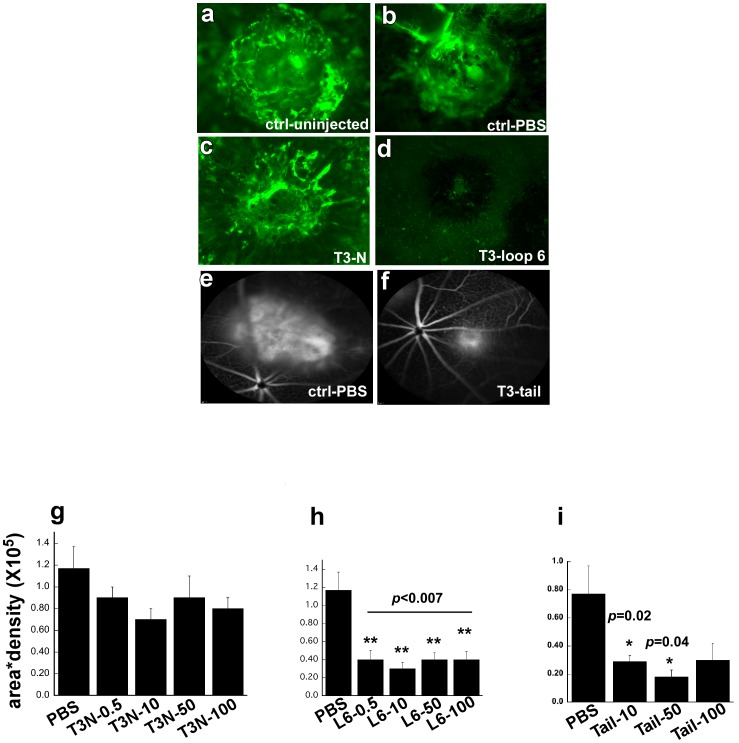
Inhibition of laser-induced CNV by TIMP-3 peptides. TIMP-3 loop 6 and tail but not N-peptide inhibit laser-induced CNV in a mouse model. Representative images of mouse choroids at 14 days following laser photocoagulation in C57BL6 mice (a) untreated/uninjected (b) injected with PBS control (c) T3N (d) T3-Loop6 (e) injected with PBS contro (f) T3-tail. Effect of Tail peptides on CNV was evaluated by Fluorescein angiogram and Scanning Laser Ophthalmoscopy (SLO). Peptides were injected intravitreally immediately following laser burn. CNV lesion size (area X density) was calculated for each dose of T3N-peptide (Fig. 7g), Loop6-peptide (Fig. 7h) and Tail-peptide (fig. 7i).

CNV was induced in mice following laser injury of Bruch’s membrane. In order to determine if TIMP-3 peptides could inhibit CNV we performed a single intravitreal injection of TIMP-3 peptides at various doses immediately following the laser burn. Mice were analyzed for the development of choroidal neovascular membranes 14 days later. Quantitative image analysis determined that T3-loop 6 (Fig. 7d) could significantly inhibit the area of CNV in mice (Fig. 7h) when compared with the CNV lesions from uninjected (Fig.7a), PBS (Fig. 7b,7e), T3-loop5 (data not shown) or T3-N peptides (Fig. 7c, g) injected animals. T3-tail peptides (Fig. 7f, 7i) showed a similar trend with 2 doses (10 µg and 50 µg) but not at the 100 µg dose (Fig. 7i). The effect of the tail peptides on laser-induced CNV was evaluated by fluorescein angiogram and scanning laser ophthalmoscopy with similar results (Fig. 7e, 7f, 7i). Injection of peptide alone in the absence of laser burn did not induce any pathology in the retina (data not shown).

## Discussion

The sprouting of new blood vessels from pre-existing vasculature, termed angiogenesis, contributes significantly to a number of pathological conditions such as cancer, diabetes and age-related macular degeneration. This process of neovascularization is kept in check by the tight regulatory balance between pro- and anti-angiogenic factors. The extracellular matrix (ECM) plays a critical role in the regulation of angiogenesis with matrix degrading enzymes having been shown to generally be pro-angiogenic and endogenous MMP inhibitors (TIMPs), anti-angiogenic. However, MMP-mediated controlled proteolysis of the ECM, also releases protein fragments such as endostatin, canstatin, tumstatin, and endorepellin that are biologically active and potent angiogenesis inhibitors [10], [19]. For a number of years, the tumor-inhibitory and anti-angiogenic properties of TIMPs were believed to be entirely due to their MMP inhibitory properties. As a consequence, there has been a considerable investment of resources to develop safe and effective therapeutic modalities that target MMPs. Several generations of synthetic MMP inhibitors were tested in phase III clinical trials in humans but were found to be surprisingly ineffective relative to the results obtained in pre-clinical trials [20].

**Figure 8:**
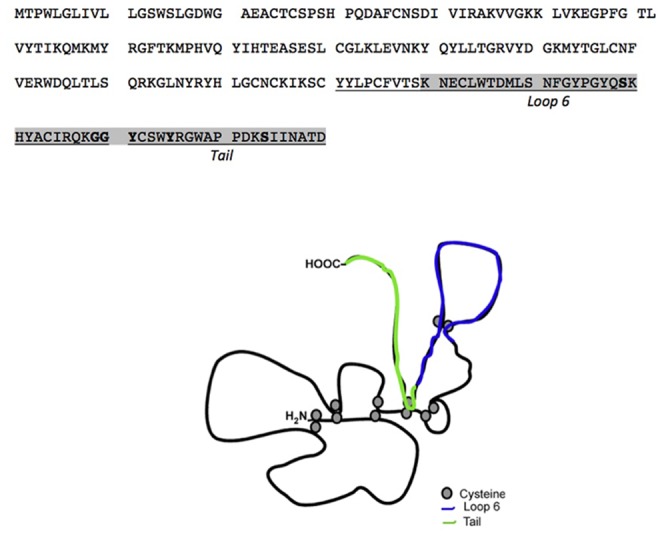
Location of Loop 6 and Tail TIMP-3 peptides on TIMP-3 protein.

More recently, TIMPs have been shown to be multifunctional proteins with a number of biological activities that were independent of their MMP inhibitory properties [7]. Inhibition of angiogenesis by TIMP-2 and TIMP-3 has been demonstrated to be independent of their ability to inhibit MMPs [8], [21], [22]. Previously described studies of the structure-functional analyses of TIMP-2 revealed that the anti-angiogenic activity of TIMP-2 was present in the C-terminal end specifically in a smaller, 2.9 kDa domain in this region (Loop 6) [21]. Based on these studies we designed experiments to determine the region of TIMP-3 that was responsible for angiogenesis inhibition. In the present study we identified the C-terminal region of the protein to be responsible for this effect, using the property of TIMP-3 to block binding of VEGF to VEGFR-2 [8]that we had previously reported. Short peptides were designed based on the functional domains of TIMP-1 [23]. We mapped the anti-angiogenic activity of TIMP-3 to peptides in the putative Loop 6 and Tail regions of the protein (Figure 8). Peptides based on the Loop 5 and N-terminal domains had no angio-inhibitory activity in vitro or in vivo. Concomitantly, Loop6 and Tail peptides were effective in inhibiting laser-induced CNV in vivo.

Sorsby fundus dystrophy (SFD), an autosomal dominant, fully penetrant, degenerative disease of the macula [24], [25] is manifested by symptoms of night blindness or sudden loss of acuity, usually in the third to fourth decades of life due to submacular neovascularization [26], [27], [28], [29]. SFD is caused by specific mutations in the tissue inhibitor of metalloproteinases-3 (*TIMP-3*) gene [30], [31], [32], [33], [34], [35], [36], [37], most of which introduce an unpaired cysteine at the C-terminus of the protein. We have recently reported that S156C mutation in TIMP-3 induces increased angiogenesis [18] and that mice lacking TIMP-3 show increased laser-induced CNV [38]. Since the anti-angiogenic activity of TIMP-3 (and TIMP-2) [21] lies in the C-terminus region of the protein and most of the new cysteines in SFD mutations lie in the same region (Fig. 8) we hypothesized that Loop 6 and Tail peptides of TIMP-3 might be critical determinants of this inhibitory activity. Whether the free cysteine in the tail peptide sequence is critical for the angiogenesis inhibition will be an interesting question to address in future studies. Sequence comparison and alignment between a short peptide sequence of pigment epithelial growth factor (PEDF) that shows anti-angiogenic activity and TIMP-3 shows a short consensus sequence of SNFGYXXY between the two proteins [39]. Both PEDF and TIMP-3 are anti-angiogenic proteins whose peptides have been shown to play a critical role in inhibiting ocular angiogenesis especially choroidal neovascularization. Finding consensus sequences in these peptides might provide clues regarding the mechanisms of inhibition of neovascularization.

VEGF plays an important role in the maintenance and function of the adult retina neuronal cells [40]. KDR has been demonstrated to be involved mainly in pathological angiogenesis. Immunohistochemical localization studies of VEGF receptors in the retina have determined VEGFR-2 to be expressed minimally in normal retina and significantly increased in both intra-and pre-retinal vessels in PDR tissue [41]. This same study showed that VEGFR-1 was present in normal retina and confined to the inner nuclear layer, ganglion cell layer and retinal vessels and significantly increased in diabetic retinas. While the exact function of VEGFR-1 has not been elucidated it has been postulated to play a role in endothelial cell homeostasis. Thus inhibiting signaling via VEGFR-1 may have a detrimental effect on the normal physiological function of endothelial cells. Thus a selective agent that can block VEGF signaling exclusively via the VEGFR-2 may have less long-term toxicity issues than a broad spectrum VEGF inhibitor.

Alanine scanning mutagenesis identified residues in the loop III region formed by the anti-parallel β sheets in VEGF protein to be responsible for binding to VEGFR-2. Blocking of VEGFR-ligand interaction has been a validated approach in drug development for CNV, as seen with the clinical success of bevacizumab (Avastin) and ranibizumab (Lucentis) in AMD. While the exact molecular mechanism(s), by which TIMP-3 peptides inhibit VEGF-mediated neovascularization have not yet been elucidated, they appear to be good candidates for drug design as they demonstrate remarkable specificity for inhibition of VEGF binding to VEGFR-2 with no effect on its binding to VEGFR-1.
